# Solid-State Fermentation of Black Gram: Improvement of Protein Digestibility and Functional Properties for Composite Flour Applications

**DOI:** 10.3390/foods15142559

**Published:** 2026-07-21

**Authors:** Pardeep Kumar Sadh, Nikhil Dnyaneshwar Patil, Neetu Mundalia, Aarti Bains, Mansuri M. Tosif, Ravinder Kaushik, Prince Chawla

**Affiliations:** 1 Department of Biotechnology, Chaudhary Devi Lal University, Sirsa 125055, Haryana, India; pardeep.sadh@gmail.com (P.K.S.); neetu03492@gmail.com (N.M.); 2 Department of Biotechnology, Graphic Era (Deemed to be University), Dehradun 248002, Uttarakhand, India; 3 Department of Food Technology and Nutrition, Lovely Professional University, Phagwara 144411, Punjab, India; nikhil2898p@gmail.com; 4 School of Health Sciences and Technology, University of Petroleum and Energy Studies, Dehradun 248007, Uttarakhand, India; aarti05888@gmail.com (A.B.); ravinder_foodtech2007@rediffmail.com (R.K.); 5 Department of Food Technology, Vocational School of Technical Sciences at Mersin Tarsus Organized Industrial Zone, Tarsus University, 33100 Mersin, Turkey; tosifmansuri444@gmail.com

**Keywords:** amino acid, mineral content, functional properties, fermentation

## Abstract

This study evaluated the effect of solid-state fermentation (SSF) using *Aspergillus awamori* on the nutritional, functional, antioxidant, structural, and protein-digestibility properties of black gram (*Vigna mungo*) flour and its application in wheat-based composite flour. Black gram was fermented for 0–12 days, and the 6-day fermented sample was selected for flour fortification based on improved nutritional and functional responses. SSF reduced tannin and phytic acid contents while increasing total phenolic content from 2.69 to 3.37 mg GAE/g, total flavonoid content from 0.83 to 1.65 mg QE/g, and DPPH radical-scavenging activity from 2.13 to 4.09 mg AAE/g by day 6. Protein content increased from 24.14% to 27.11%, and in vitro protein digestibility improved from 70.86% to 82.89%. Total amino acid content also increased from 88.71 to 98.24 g/100 g protein by day 6, followed by a decline during prolonged fermentation. Mineral contents, including iron, zinc, and calcium, were also improved after fermentation. Incorporation of 6-day fermented black gram flour into wheat flour enhanced the protein, crude fiber, water-holding capacity, oil-holding capacity, and in vitro protein digestibility of the composite flour. Overall, SSF with *A. awamori* improved the nutritional and functional quality of black gram and demonstrated its potential as a protein-rich ingredient for fortified cereal–legume composite flour development.

## 1. Introduction

The global population is projected to reach approximately 10 billion by 2050, presenting substantial challenges for ensuring adequate food, nutrition, and health security [1]. Malnutrition, particularly undernutrition, is largely due to limited access to nutrient-dense foods, including protein and specific micronutrients [2]. Adequate nutrition is fundamental for maintaining health and development of babies, children and mothers [3]. Inadequate intake of protein and essential micronutrients during childhood results in underweight, muscle protein breakdown, impaired linear growth (stunting), and protein-energy malnutrition (PEM) [4]. Therefore, there is an urgent need to diversify food sources and develop functional foods using underutilized crops with superior nutritional profiles and adaptability to diverse agro-climatic conditions. Recent projections indicate that global demand for animal-derived protein will nearly double by 2050, driving the need for sustainable, plant-based protein innovations that can complement or replace animal-derived sources while minimizing greenhouse gas emissions and freshwater use [5,6]. A wide variety of plant-derived protein sources are available, encompassing cereals (wheat, rice, millet, maize, barley, and sorghum), legumes (pea, soybean, bean, faba bean, lupin, chickpea, and cowpea), pseudocereals (buckwheat, quinoa, and amaranth), together with nuts and seeds including flaxseed, chia, pumpkin, sesame, and sunflower [7].

Black gram (*Vigna mungo* L.), commonly referred to as “urd” in India, is one of the most important leguminous pulses cultivated in South Asia [8]. It is a rich source of protein (24%) and carbohydrates (59.6%) and provides substantial amounts of essential minerals, including calcium, phosphorus, and iron, as well as B-complex vitamins [9]. Black gram proteins are a good source of essential amino acids, particularly lysine and tryptophan, compared with mung bean, pigeon pea, lupin, and moth bean [10]. Beyond its nutritional profile, black gram possesses techno-functional properties such as water absorption capacity, water solubility index, and oil absorption capacity [11]. Legumes contain various antinutritional factors, such as tannins and phytic acid, that limit digestion, reduce nutrient absorption, and inhibit metabolism [12]. Therefore, appropriate processing strategies are essential to reduce these antinutritional compounds, thereby improving protein digestibility, mineral bioavailability, and the overall nutritional quality of legumes.

Fermentation represents one of the oldest bioprocessing techniques for improving the nutritional, and sensory attributes of foods. It is widely used to ferment cereals and legumes, two of the world’s most important staple crops, producing a broad range of food products [13]. Fermentation can be carried out through spontaneous, back-slopping, or controlled approaches. Spontaneous fermentation is driven by the sequential succession of naturally occurring microorganisms, during which the best-adapted microbial populations proliferate and ultimately dominate the fermentation ecosystem. In contrast, back-slopping fermentation involves inoculating a fresh substrate with a small portion of a previously fermented batch, thereby facilitating the transfer of beneficial microorganisms and improving fermentation consistency. Controlled fermentation, however, employs well-defined starter cultures, such as lactic acid bacteria, yeasts, fungi, or *Bacillus* spp., to achieve greater process control, and reproducibility, and product quality [13,14,15,16].

Furthermore, fermentation is broadly categorized into two principal types: solid-state fermentation (SSF) and submerged fermentation (SmF). SmF involves the cultivation of microorganisms in a liquid nutrient medium, whereas SSF is performed on moist solid substrates with minimal or no free water [17]. Compared with submerged fermentation, Solid-state fermentation (SSF) has developed significantly over the last two decades, and its advantages generally outweigh the engineering challenges associated with the process. It can produce higher amounts of value-added products than submerged fermentation. At the same time, the use of bacterial cultures reduces fermentation time and capital costs, and an inert support provides favorable fermentation conditions and improves product purity. However, SSF also has several limitations, including difficulties with scale-up, controlling heat generation, directly estimating biomass, and handling heterogeneous fermentation conditions. Therefore, further improvements in bioreactor design, process control, substrate optimization, and heat transfer are required for efficient large-scale applications [18,19,20].

Although previous studies have reported the effects of solid-state fermentation on selected nutritional and functional attributes of black gram, limited information is available on the simultaneous evaluation of its biochemical composition, protein quality indices, mineral content, antioxidant potential, and application in composite flour systems. Therefore, the objective of this study was to evaluate the biochemical and nutritional modifications in black gram subjected to SSF, focusing on changes in nutrient composition, bioactive compounds, protein and amino acid profiles, mineral content, and in vitro protein digestibility. Furthermore, the fermented black gram was incorporated into a composite flour system to assess its functional properties and protein digestibility within a practical food matrix. This application-based evaluation provides additional insight into the potential use of fermented black gram in fortified cereal–legume blends and other value-added functional food formulations. The findings contribute to the growing body of knowledge on sustainable processing approaches for developing nutritionally improved legume-based functional foods with potential applications in addressing protein malnutrition.

## 2. Materials and Methods

### 2.1. Materials

Seeds of black gram were sourced from Krishi Vigyaan Kendra, Sirsa, Haryana, and maintained at ambient temperature in airtight containers until analysis. At the same time, all analytical-grade reagents and chemicals were supplied by HiMedia Laboratories Pvt. Ltd. (Mumbai, India). Borosilicate glassware was used in all experiments and was thoroughly washed with detergent and triple-distilled water before use.

### 2.2. Methods

#### 2.2.1. Proximate Analysis of Black Gram

The proximate composition of legume powders, including black gram, was assessed using standardized AOAC procedures [21]. Moisture and ash content were measured gravimetrically, while crude protein was quantified using the Kjeldahl technique with a conversion factor of 6.25. Crude fiber was determined via acid–alkali digestion, and crude fat was extracted through the Soxhlet method. Carbohydrate content was calculated by difference, subtracting the combined percentages of moisture, protein, fat, and ash from 100.

#### 2.2.2. Substrate Preparation

The substrate was initially rinsed thoroughly with tap water to remove surface contaminants, then air-dried for 6 h under ambient conditions. After drying, the seeds were used directly in the experiments without any additional processing.

#### 2.2.3. Microorganism Used and Inoculum Preparation

The culture was obtained from the MTCC, Institute of Microbial Technology, Chandigarh, India. For propagation, the fungus was maintained on potato dextrose agar (PDA) and routinely subcultured onto fresh PDA plates. Incubation was performed at 25 °C for 6 days. Spore suspensions were prepared in sterile, cell-grade water, achieving a final concentration of approximately 1 × 10^6^ spores/mL. The PDA formulation included peeled potato infusion (200 g/L), dextrose (20 g/L), and agar (15 g/L), with the pH adjusted to 5.6 ± 0.20. Czapek–Dox medium, prepared at pH 7.3 ± 0.20, was also used for fungal growth and contained sodium nitrate (2.5 g/L), monopotassium phosphate (1.0 g/L), potassium chloride (0.5 g/L), and magnesium sulfate heptahydrate (0.5 g/L).

#### 2.2.4. Fermentation of Black Gram

**Figure 1:**
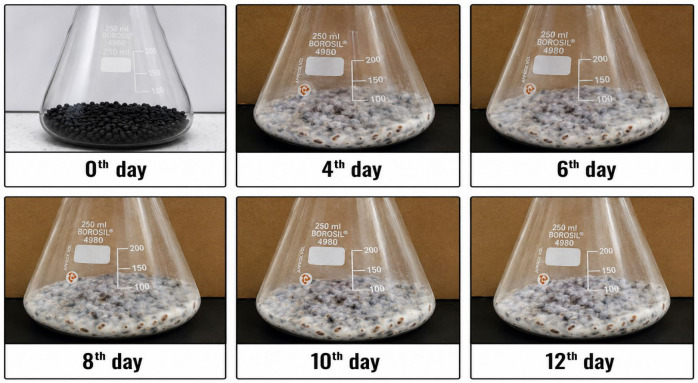
Fermentation of black gram using *Aspergillus awamori* over 12 days (0, 4, 6, 8, 10, and 12 days).

Solid-state fermentation of black gram was conducted as described by Dhull et al. [22], with minor modifications. Whole black gram seeds with intact seed coats were used throughout the study; no dehulling was performed. For each fermentation unit, 25 g of black gram seeds were placed in a 250 mL Erlenmeyer flask and soaked overnight at 30 °C in 75 mL of Czapek–Dox medium. After soaking, the excess medium was decanted, and the moisture content of the hydrated substrate was adjusted to 60% (wet basis) before sterilization. The hydrated substrate was sterilized at 121 °C and 15 psi for 15 min and allowed to cool to room temperature under aseptic conditions. Thereafter, 2.5 mL of *Aspergillus awamori* spore suspension (1 × 10^6^ spores/mL) was added to each flask and mixed manually to ensure uniform distribution of the inoculum. The inoculated flasks were incubated at 30 °C in a BOD incubator for 0, 4, 6, 8, 10, and 12 days. Fermentation was carried out under static conditions. No daily shaking, turning, or manual mixing was performed during incubation. No forced aeration was applied; passive gas exchange was maintained through the flask headspace and sterile cotton-plug closure. Separate fermentation flasks were used for each incubation period, and samples were withdrawn at the respective sampling times for further analysis as shown in Figure 1.

#### 2.2.5. Determination of Tannins and Phytic Acid Content

Tannin content was determined according to the protocol of Gupta et al. [23], with slight modifications. Briefly, 400 mg of the sample was extracted with 40 mL of distilled water. The mixture was boiled for 30 min, allowed to cool to room temperature, and centrifuged at 2000× *g* for 10 min. The resulting clear supernatant was collected for analysis. An aliquot of 1 mL of the extract was mixed with 5 mL of Folin–Denis reagent and 10 mL of sodium carbonate solution. The reaction mixture was then diluted to a final volume of 100 mL with distilled water and incubated at room temperature for 30 min to allow color development. The absorbance was measured at 700 nm using a UV–Visible spectrophotometer (Systronics-168, Systronics India Ltd., Ahmedabad, India), and tannin content was expressed as tannic acid equivalents.

Phytic acid content was determined according to the protocol described by Gupta et al. [23]. Briefly, 50 mg of the finely powdered sample was extracted overnight in 0.4 mM hydrochloric acid (HCl) at room temperature. The extract was subsequently centrifuged at 10,000× *g* for 20 min, and the supernatant was collected for analysis. For the assay, 10 μL of the extract was transferred to a microtiter plate and diluted with 90 μL of double-distilled water. Subsequently, 100 μL of freshly prepared colorimetric reagent, containing 3 M sulfuric acid (H_2_SO_4_), 2.5% ammonium molybdate, 10% (*w*/*v*) ascorbic acid, and distilled water in a 1:1:1:2 ratio, was added to each well. The reaction mixture was incubated at room temperature for 60 min, after which the absorbance was recorded at 650 nm using a UV–Visible spectrophotometer.

#### 2.2.6. Total Phenolic Content

The ethanolic extract was prepared to determine total phenolic and flavonoid content and DPPH radical-scavenging activity. Briefly, 1 g of finely ground black gram flour from each fermentation interval was mixed with 20 mL of 80% ethanol in a conical flask. The mixture was vortexed thoroughly, then placed on a shaker at 150 rpm for 2 h at room temperature to facilitate the extraction of antioxidant compounds. After extraction, the suspension was centrifuged at 5000× *g* for 10 min, and the clear supernatant was collected. The remaining residue was re-extracted once under the same conditions using 20 mL of 80% ethanol to improve extraction efficiency. Both supernatants were pooled and filtered through Whatman No. 1 filter paper. The final ethanolic extract was stored at 4 °C and used within 24 h for TPC, TFC, and DPPH analysis. For the DPPH assay, the extract was suitably diluted to obtain a working concentration of 1 mg/mL. The total phenolic contents were quantified following the method of Phuyal et al. [24], with slight modifications to suit the experimental conditions. Briefly, appropriately diluted sample extracts (25–100 μg/mL) were mixed with 5 mL of 10% (*v*/*v*) Folin–Ciocalteu reagent and 4 mL of 7% (*w*/*v*) sodium carbonate solution to obtain a final volume of 10 mL. The reaction mixture was thoroughly mixed and incubated at 40 °C for 30 min. Absorbance was then measured at 760 nm using a UV–Vis spectrophotometer against a reagent blank. Gallic acid (25–100 μg/mL) was used as the standard for preparing the calibration curve. The total phenolic content was calculated from the standard curve and expressed as milligrams of gallic acid equivalents per gram of dry sample (mg GAE/g dry weight). All measurements were performed in triplicate, and the results were expressed as mean ± standard deviation.

#### 2.2.7. Total Flavonoid Content

The total flavonoid contents were quantified following the method of Phuyal et al. [24], with slight modifications to suit the experimental conditions. Briefly, appropriately diluted sample extracts (0.25–1.0 mg/mL) were mixed with 4 mL of distilled water and 0.3 mL of 5% (*w*/*v*) sodium nitrite solution. After 5 min, 0.3 mL of 10% (*w*/*v*) aluminum chloride solution was added. Six minutes later, 2 mL of 1 M sodium hydroxide was added, and the final volume was adjusted to 10 mL with distilled water. The mixture was thoroughly mixed, and the absorbance was measured at 510 nm using a UV–Vis spectrophotometer. Quercetin (0.25–1.0 mg/mL) was used as the standard for preparing the calibration curve. Total flavonoid content was calculated from the standard curve and expressed as milligrams of quercetin equivalents per gram of dry sample (mg QE/g dry weight).

#### 2.2.8. DPPH Assay

The DPPH radical-scavenging activity was determined according to Sombié et al. [25], with minor modifications. A freshly prepared 0.1 mM DPPH solution was used as the working reagent. The sample extract was prepared at a concentration of 1 mg/mL (*w*/*v*). Briefly, 100 µL of the sample extract was mixed with 3.0 mL of 0.1 mM DPPH solution, resulting in a total reaction volume of 3.1 mL. The mixture was vortexed and incubated at 37 °C in the dark for 30 min. Thereafter, absorbance was measured at 517 nm using a UV–Visible spectrophotometer. The control contained 100 µL of the extraction solvent in place of the sample extract, together with 3.0 mL of DPPH solution. A reagent blank containing the respective solvent without DPPH was used to correct the instrument baseline. Ascorbic acid was used as the reference standard, and the antioxidant activity of the samples was expressed as mg ascorbic acid equivalents (AAE)/g dry extract.

#### 2.2.9. Characterization of Powder

##### Amino Acid Content

The amino acid profiles of various legume powders were analyzed using the method described in Patil et al. [26]. Amino acid analysis was conducted by hydrolyzing 10 mg of sample powder in 20 mL of 5 M hydrochloric acid, followed by the addition of phenol crystals. The mixture was vacuum-dried at 110 °C for 25 h. Derivatization was performed using a borate buffer (pH 8.2–10.0) and the Accq-Fluor reagent kit (WAT052890, Waters Millipore Corporation, Billerica, MA, USA, 1993), according to the manufacturer’s protocol. Quantification was performed by high-performance liquid chromatography with fluorescence detection (HPLC-FLD, Eurofins, Bengaluru, India) using a binary gradient system and an AccQ-Tag Nova-Pak C18 column (3.8 × 151 mm, 6 µm silica). Quantification was performed using external calibration with a mixture of 16 amino acid reference standards, in accordance with the accredited laboratory’s validated analytical protocol.

##### Fourier-Transform Infrared Spectroscopy

Fourier-transform infrared spectroscopy (FTIR) was employed to characterize the functional groups present in the sample using both attenuated total reflectance (ATR) and KBr pellet techniques.

#### 2.2.10. Functional Properties

##### Protein Content

Protein content in the powdered samples was quantified using the Lowry assay, as per the modified protocol outlined by Patil et al. [26].

##### Water and Oil Holding Capacity

Water holding capacity (WHC) and oil holding capacity (OHC) were measured following the method of Sharma et al. [27]. A 1 g portion of the powdered sample was thoroughly blended with 15 mL of either deionized water or soybean oil and then incubated for 30 min. Following incubation, the mixture was centrifuged at 6000× *g* for 10 min. The retained liquid was calculated by the weight difference and expressed as grams of liquid per gram of sample.

##### Emulsion Capacity and Stability

The emulsifying ability (EA) and emulsion stability (ES) of the sample were evaluated based on the modified protocol described by Qadir et al. [28]. For this, a 1% (*w*/*v*) aqueous dispersion of the powder was homogenized with 1 mL of canola oil at 5000 rpm for 30 min to form a stable emulsion. The mixture was then centrifuged at 10,000× *g* for 20 min to facilitate phase separation. EA and ES were calculated using the following standard Equations (1) and (2).

(1) $$\mathrm{Emulsion}\;\mathrm{capacity}\;(\%)=\left\{\left(\frac{Height\;of\;the\;emulsified\;phase}{Total\;height}\right)\times 100\right\}$$

To determine emulsion stability, the emulsion was subjected to thermal treatment at 70 °C for 30 min, followed by centrifugation at 6000× *g* for 15 min. The remaining emulsified layer post-heating was used to calculate ES as follows:

(2) $$\mathrm{Emulsion}\;\mathrm{stability}\;(\%)=\left\{\left(\frac{Height\;of\;the\;Emulsified\;layer\;after\;heating}{Height\;of\;the\;emulsified\;layer\;before\;heating}\right)\times 100\right\}$$

##### Foaming Capacity and Stability

Foaming properties, including foaming capacity (FC) and foam stability (FS), were determined using a modified method based on Ozolina et al. [29], with minor changes. In brief, 0.5 g of the sample powder was suspended in 25 mL of deionized water and homogenized by continuous stirring for 15 min. The resulting dispersion was transferred into a 250 mL graduated measuring cylinder to assess foam formation. Foaming capacity was calculated by measuring the increase in volume immediately after whipping, using the following Equations (3) and (4).

(3) $$Foaming\;capacity\left(\%\right)=\left[\left(\frac{Volume\;after\;whipping\left(mL\right)-Volume\;before\;whipping\;(mL)}{Volume\;before\;whipping\;(mL)}\right)\times 100\right]$$

Foam stability was assessed by allowing the foam to stand undisturbed for 30 min at 25 ± 2 °C, after which the remaining foam volume was recorded. The percentage of foam retained was calculated using the following equation.

(4) $$Foaming\;stability\;(\%)=\left[\left(\frac{Volume\;after\;standing\;time\left(mL\right)-Volume\;before\;whipping\;(mL)}{Volume\;before\;whipping\;(mL)}\right)\times 100\right]$$

#### 2.2.11. Mineral Content

The levels of calcium, zinc, and iron in the powdered samples were quantified using Atomic Absorption Spectrophotometry (AAS) in accordance with the standardized procedures outlined by Chawla et al. [30]. For analysis, samples were incinerated at 550 °C for 8 h, then acid-digested with a tri-acid mixture. The digests were then heated until complete dissolution occurred, and the resulting solutions were appropriately diluted before AAS quantification.

#### 2.2.12. In Vitro Digestibility

In vitro protein digestion was carried out following the internationally standardized static gastrointestinal digestion protocol described by Benhammouche et al. [31]. The digestion procedure simulated the oral, gastric, and intestinal phases of human digestion. Briefly, 1 g of each sample (MODL and PC) was mixed with 4 mL of simulated salivary fluid (SSF), 0.5 mL of α-amylase solution (1500 U/mL prepared in SSF), 25 µL of 0.3 M CaCl_2_, and 475 µL of ultrapure water. The oral digestion mixture was incubated for 2 min at 37 °C. Following the oral phase, the entire mixture (10 mL) was combined with 8 mL of simulated gastric fluid (SGF), 5 µL of 0.3 M CaCl_2_, and sufficient 1 M HCl or ultrapure water to adjust the pH to 3.0, resulting in a total added volume of 1.395 mL. Subsequently, 0.5 mL of pepsin solution (25,000 U/mL prepared in SGF) was added, and the gastric digestion mixture was incubated at 37 °C for 2 h with continuous agitation. After gastric digestion, 20 mL of the gastric digest was mixed with 85 mL of simulated intestinal fluid (SIF) electrolyte stock solution and 40 µL of 0.3 M CaCl_2_. The pH was adjusted to 7.0 using 1 M NaOH or ultrapure water, yielding a final added volume of 3.76 mL. Thereafter, 5 mL of pancreatin solution (800 U/mL in SIF, based on trypsin activity) and 2.5 mL of bile salt solution (160 mM) were added. The intestinal digestion mixture was incubated at 37 °C under continuous mixing. All digestion experiments were performed in triplicate. Parallel assays were conducted to determine the volume of acid or base required to adjust the pH at each digestion stage. Enzyme blanks containing only the digestive fluids and enzymes were prepared simultaneously, and all analytical results were corrected using the corresponding blank values. Upon completion of the intestinal digestion, the reaction mixtures were immediately cooled in an ice bath and centrifuged at 5000× *g* for 10 min at 4 °C to separate the soluble bioaccessible fraction from the undigested residue. The collected supernatants, representing the bioaccessible protein fraction, were stored at −20 °C until amino acid analysis. The bioaccessible protein content was calculated by subtracting the amount of undigested protein remaining in the pellet after centrifugation from the sample’s initial protein content. Subsequently, the in vitro protein digestibility (%) was determined by dividing the bioaccessible protein content by the sample’s initial protein content and multiplying the result by 100.

#### 2.2.13. Preparation of Black Gram Powder Fortified Flour

Whole-wheat flour was combined with fermented black gram powder at substitution levels of 15% and 20% (*w*/*w*). These substitution levels were selected to evaluate the effect of moderate incorporation of the fermented black gram powder into whole-wheat flour while maintaining a practical level of flour replacement. The blends were mechanically mixed for 1 min using a stand mixer (Model 5K45SS Heavy Duty, KitchenAid, Greenville, OH, USA) to achieve uniform dispersion of the protein-enriched fraction (Sachanarula et al. [32]).

#### 2.2.14. Proximate Analysis of Black Gram Powder Fortified Flour

Proximate constituents were quantified in accordance with established AOAC protocols [33,34]. Moisture, crude protein, ash, crude fiber, and lipid contents were evaluated. Nitrogen was measured by the micro-Kjeldahl procedure and converted to protein using the appropriate factor. Crude fiber was determined by consecutive acid and alkaline digestion, while total lipids were extracted using the Soxhlet technique. The carbohydrate fraction was calculated by difference as follows: 100% − (protein + moisture + ash + fat).

#### 2.2.15. Water- and Oil-Holding Capacity of the Fortified Flour

The capacities of flour to retain water and oil were assessed based on the method described by Ma et al. [35], with minor procedural adjustments. The detailed methodology has been written in Section 2.2.10 Functional Properties.

#### 2.2.16. Water Solubility Index of Fortified Flour

Water solubility of the flour was evaluated following the protocol reported by Du et al. [36], with minor adjustments. One gram of the sample was dispersed in 10 mL of distilled water in a 15 mL centrifuge tube and incubated in a water bath at 37 °C for 30 min. The suspension was centrifuged at 4000 rpm for 10 min, after which the supernatant was transferred to a pre-weighed vessel and oven-dried at 105 °C until a constant mass was achieved. The residue recovered from the supernatant represented the soluble fraction. The WSI was calculated as the percentage of this dried soluble material relative to the initial sample mass, as given in Equation (5).

(5) $$\mathrm{WSI}\;(\%)=\frac{Weight\;of\;dried\;supernatant}{Dry\;weight\;of\;sample}\times 100$$

#### 2.2.17. In Vitro Protein Digestibility of Flour

In vitro digestion was carried out following the standardized protocol outlined by Benhammouche et al. [31]. This procedure included three sequential phases: an oral phase using α-amylase, a gastric phase using pepsin in hydrochloric acid, and an intestinal phase involving pancreatin and bile salts to simulate human digestion. The section has been fully written in Section 2.2.11.

#### 2.2.18. Statistical Analysis

All experimental measurements were carried out in triplicate, and the results are presented as mean ± standard deviation (*n* = 3). Three independently prepared and inoculated fermentation flasks were used for each fermentation time point. Each analytical determination was performed in triplicate for each biological replicate, and the reported values represent the mean ± standard deviation of three independent fermentation replicates. Statistical analyses were performed using one-way analysis of variance (ANOVA), followed by Duncan’s post hoc test, at a significance level of *p* < 0.05 to compare multiple fermentation time points. In contrast, independent *t*-tests were used only for comparisons between two groups using IBM SPSS Statistics 27 (IBM Corp., Armonk, NY, USA). Differences were considered statistically significant at *p* < 0.05.

## 3. Results and Discussion

### 3.1. Proximate Analysis of Black Gram

The proximate composition of black gram is presented in Table 1. The protein content was 24.13 ± 0.25%**,** indicating that black gram is a good source of plant protein. The carbohydrate content was the highest among all measured components, accounting for 55.53 ± 0.57%**,** followed by moisture (10.49 ± 0.08%). The crude fiber, ash, and fat contents were 5.13 ± 0.16%, 3.28 ± 0.17%, and 1.44 ± 0.05%, respectively. Our results are similar to those of Wani et al. [37], in which the protein content is comparable to values reported for black gram in previous studies, generally ranging from 24.5 to 28.4%**.** Similarly, the moisture (10.3 to 11.6%), fat (1.1 to 1.4%), ash (2.7 to 3.3%), and carbohydrate (54.1 to 56.5%) contents were observed. Likewise, Soharu et al. [38] reported protein, moisture, fat, ash, dietary fiber, and carbohydrate contents of 24.24 to 28.22%, 11.2 to 11.7%, 1.25 to 1.85%, 3.10 to 4.45%, 5.35 to 6.60%, and 60.23 to 64.86%, respectively, among different black gram genotypes. The slight differences between the present findings and the reported values may be attributed to differences in cultivar, growing conditions, and environmental factors. Overall, the proximate composition obtained in the present study is consistent with values reported in the literature, indicating that the black gram sample used had a typical nutritional composition.

### 3.2. Tannin and Phytic Acid Content of Black Gram

**Figure 2:**
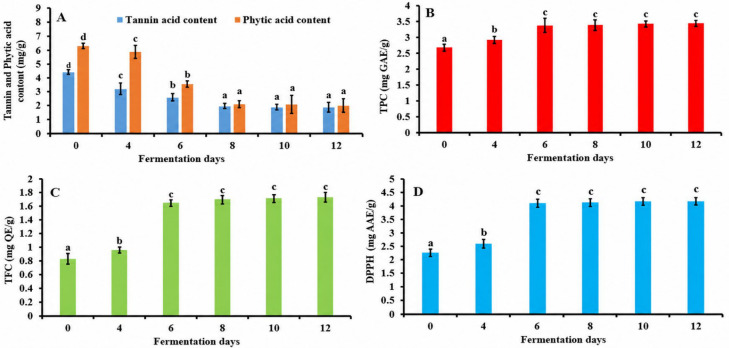
(**A**) Tannin and phytic acid content; (**B**) total phenolic content (TPC); (**C**) total flavonoid content (TFC); and (**D**) DPPH of black gram fermented using *Aspergillus awamori* over 12 days (0, 4, 6, 8, 10, and 12 days). The results were expressed as the mean ± standard deviation (*n* = 3), and error bars represent the standard deviation from the mean values. In contrast, different lowercase letters above each bar indicate significantly different values (*p* < 0.05) within samples, as determined by one-way ANOVA followed by Duncan’s post hoc test for multiple comparisons.

Figure 2A illustrates the variations in tannin and phytic acid concentrations observed during the fermentation of black gram with *Aspergillus awamori*. A substantial reduction in both anti-nutritional factors was observed. Initially, the tannin content in the native (unfermented) black gram was 4.45 mg/g. It declined significantly (*p* ≤ 0.05) to 1.88 mg/g by day 12 of fermentation, representing a reduction of approximately 57.75%. Similarly, phytic acid content decreased from 6.32 ± 0.23 to 2.01 ± 0.49 mg/g, corresponding to a reduction of approximately 68.19% after 12 days of fermentation. A similar decreasing trend in phytic acid during fermentation has been reported by Yadav & Khetarpaul [39], who observed significant reductions of phytic acid in black gram with increasing fermentation time. Similarly, the decrease in tannin content during fermentation may be attributed to the production of tannase, which hydrolyzes tannins into simpler phenolic compounds [40]. Overall, solid-state fermentation with *Aspergillus awamori* effectively reduced tannin and phytic acid contents, demonstrating its potential to improve the nutritional quality of black gram.

### 3.3. Phenolic and Antioxidant Activity

#### 3.3.1. Total Phenolic and Flavonoid Content

The changes in total phenolic content (TPC) during solid-state fermentation of black gram with *Aspergillus awamori* are presented in Figure 2B. The TPC increased significantly (*p* ≤ 0.05) from 2.69 mg GAE/g in the unfermented sample to 3.37 mg GAE/g after 6 days of fermentation. After that, it increased non-significantly. The highest TPC (3.44 mg GAE/g) was observed after 12 days of fermentation, representing an increase of approximately 27.88% compared to the native sample. The increase in total phenolic content during fermentation may be attributed to the release of bound phenolic compounds from the plant cell wall matrix [41].

Similarly, the effect of solid-state fermentation on the total flavonoid content (TFC) of black gram is presented in Figure 2C. The TFC increased significantly (*p* ≤ 0.05) from 0.83 mg QE/g in the unfermented sample to 1.65 mg QE/g by the 6th day of fermentation. Statistical analysis indicated no significant difference (*p* > 0.05) among the 6-, 8-, 10-, and 12-day fermented samples. The observed increase in total flavonoid content may be attributed to fermentation-induced changes in the plant matrix, which could enhance the extractability and availability of flavonoids. The results demonstrated that solid-state fermentation positively influenced the total phenolic and flavonoid contents of black gram. A gradual increase in both bioactive compounds was observed as fermentation time increased.

#### 3.3.2. DPPH Radical Scavenging Activity

The DPPH radical-scavenging activity of black gram increased significantly during solid-state fermentation with Aspergillus awamori (Figure 2D). The unfermented black gram showed the lowest antioxidant activity, at 2.13 mg AAE/g, which increased to 2.44 mg AAE/g on day 4. A marked increase was observed by the 6th day of fermentation, where DPPH activity reached 4.09 mg AAE/g. After day 6, only slight changes were observed on days 8, 10, and 12, and these values were statistically non-significant compared with day 6. This indicates that antioxidant activity improved primarily up to the 6th day, then entered a stable phase. The enhancement in DPPH radical-scavenging activity may be linked with the increase in total phenolic and flavonoid contents during fermentation. In the present study, TPC increased from 2.69 to around 3.37 mg GAE/g, while TFC increased from 0.83 to 1.65 mg QE/g by day 6, supporting the improvement in antioxidant potential. Fermentation can release bound phenolic compounds from the seed matrix through microbial enzymatic activity, thereby increasing the availability of low-molecular-weight antioxidant compounds. Similar findings were reported by Saharan et al. [42], who observed improved DPPH radical-scavenging activity in fermented legumes, including red lentil, mung bean, pigeon pea, and cowpea, following solid-state fermentation.

Overall, the results suggest that solid-state fermentation effectively enhanced the antioxidant potential of black gram. Since the values after day 6 did not differ significantly, 6 days of fermentation may be considered sufficient to achieve maximum DPPH radical scavenging.

### 3.4. Functional Properties of Black Gram

#### 3.4.1. Protein Content

**Figure 3:**
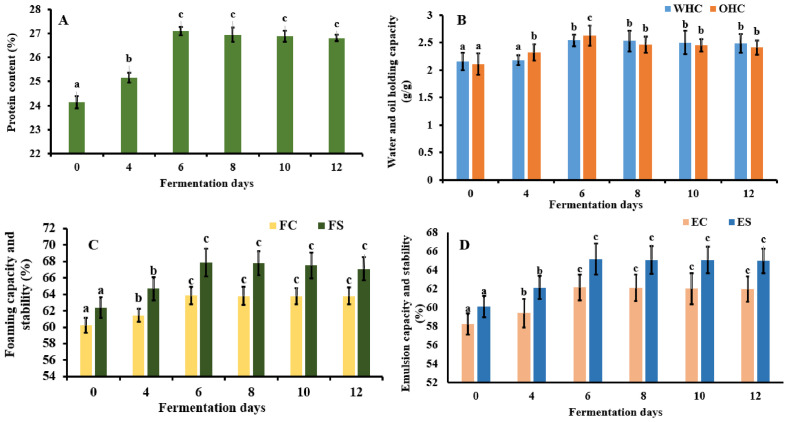
(**A**) Protein content, (**B**) water- and oil-holding capacity, (**C**) Foaming capacity and stability, and (**D**) Emulsion capacity and stability of black gram fermented using *Aspergillus awamori* over 12 days (0, 4, 6, 8, 10, and 12 days). The results were expressed as the mean ± standard deviation (*n* = 3), and error bars represent the standard deviation from the mean values. In contrast, different lowercase letters above each bar indicate significantly different values (*p* < 0.05) within samples, as determined by one-way ANOVA followed by Duncan’s post hoc test for multiple comparisons.

As shown in Figure 3A, fermentation of black gram flour with Aspergillus awamori led to a significant increase in protein content over time. The initial protein level of 24.14% (day 0) increased significantly (*p* ≤ 0.05), reaching 27.11 by day 6. After day 6, protein content was non-significantly reduced (*p* ≥ 0.05) to 26.95% (day 8), 26.89% (day 10), and 26.82% (day 12). The increase in protein content during solid-state fermentation may be associated with dry matter loss, resulting in a relative increase in protein concentration [43]. Similarly, Gautheron et al. [44] reported that the protein content of fava bean flour (FB) increased after solid-state fermentation with *Aspergillus oryzae* (FBA) and *Rhizopus oligosporus* (FBR). The protein content of FB (24.58%) increased by 20.3% and 8.1% in FBA and FBR, respectively.

#### 3.4.2. Water and Oil Holding Capacity

The oil holding capacity (OHC) and water holding capacity (WHC) of black gram flour exhibited a significant increase during solid-state fermentation by *Aspergillus awamori*, suggesting significant alterations in the physicochemical and structural properties of the macromolecules, as illustrated in Figure 3B. WHC significantly increased (*p* ≤ 0.05) from 2.16 g/g (day 0) to 2.54 g/g (day 6). In comparison, OHC rose from 2.11 g/g to 2.63 g/g in the same period. Islam et al. [45] stated that the increase in OHC and WHC may be associated with fermentation-induced degradation of starch and protein molecules, consistent with the increased protein content. Similarly, Zhao et al. [46] suggested that the improvement in water-holding capacity after fermentation may be attributed to an increase in soluble dietary fiber, which has a greater water-absorption capacity than insoluble dietary fiber. The non-significant decrease observed after day 6 suggests that the functional properties remained relatively stable during the later stages of fermentation. The findings of the present study are consistent with those of Zhao et al. [46], who reported a significant increase in the water-holding capacity of wheat bran following fermentation. From a food development perspective, increased WHC and OHC are beneficial: higher WHC improves water retention, dough handling, and product yield, while higher OHC enhances flavor retention and texture. These improvements indicate the potential of fermented black gram flour as a functional ingredient in value-added food products.

#### 3.4.3. Foaming Capacity and Stability

As depicted in Figure 3C, both foaming capacity (FC) and foaming stability (FS) of black gram flour improved notably during the early phase of fermentation with *Aspergillus awamori*. FC showed a significant increase (*p* ≤ 0.05), rising from 60.23% on day 0 to a maximum of 63.85% by day 6. Similarly, FS increased from 62.40% to 67.85% within the same timeframe. The improvement in FC and FS during fermentation may be associated with fermentation-induced modifications in protein structure, which could enhance their ability to adsorb at the air–water interface and stabilize foam. After day 6, however, both foaming capacity (FC) and foam stability (FS) reduced insignificantly (*p* ≥ 0.05). FC showed a slight reduction to 63.80% (day 8), 63.79% (day 10), and 63.78% (day 12), while FS decreased to 67.12% (day 12). The slight decline observed after day 6 may indicate that these functional changes had reached a relatively stable stage. In contrast to the present findings, Gautheron et al. [44] reported a decrease in the foaming capacity of fava bean flour following solid-state fermentation with *Aspergillus oryzae* and *Rhizopus oligosporus*. The contrasting results may be attributed to differences in legume type, microbial strain, or fermentation conditions. Improvements in FC and FS are advantageous for food applications, as these properties enhance air incorporation and foam stability, thereby enhancing texture and product quality. Therefore, fermented black gram flour shows potential for use in bakery and other aerated food products.

#### 3.4.4. Emulsion Capacity and Stability

The emulsion capacity (EC) and emulsion stability (ES) of black gram flour increased progressively during fermentation with Aspergillus awamori (Figure 3D). EC increased significantly (*p* ≤ 0.05) from 58.23% (day 0) to 62.16% on day 6. In comparison, ES improved from 60.12% to 65.16% over the same period. After day 6, there is a slight (*p* ≥ 0.05) reduction in EC (61.96% at day 12) and ES (64.99% at day 12), indicating a plateau or possible degradation of key emulsifying agents. The improvement in emulsifying properties may be associated with fungal proteolytic activity, which exposes hydrophobic groups and produces low-molecular-weight peptides that readily migrate to the oil–water interface during fermentation [30,47,48]. Our study aligns with Chawla et al. [30], in which the emulsion capacity of black-eyed flour increases significantly after fermentation.

### 3.5. Characterization of Black Gram

#### 3.5.1. Amino Acid Content

The amino acid composition of black gram during solid-state fermentation with *Aspergillus awamori* is presented in Table 2. Fermentation influenced both essential and non-essential amino acid contents throughout the 12-day fermentation period. The total amino acid content increased from 88.71 g/100 g protein in the unfermented sample to 95.74 g/100 g protein on day 4 and reached a maximum of 98.24 g/100 g protein on day 6. Thereafter, the total amino acid content gradually decreased to 93.11, 89.22, and 84.84 g/100 g protein on days 8, 10, and 12, respectively. Among the essential amino acids, histidine, isoleucine, methionine, phenylalanine, threonine, and valine showed an increasing trend up to day 6, followed by a gradual decline during the later stages of fermentation. Leucine increased from 6.50 to 7.13 g/100 g protein on day 4, remained relatively stable thereafter, and then slightly decreased by day 12. Lysine exhibited only minor variations throughout fermentation, with values ranging from 5.91 to 6.40 g/100 g protein. A similar trend was observed for the non-essential amino acids. Alanine, arginine, aspartic acid, cysteine, glycine, proline, serine, and tyrosine increased during the initial stages of fermentation, reaching their highest values around day 6, after which they gradually declined. Glutamic acid remained the most abundant amino acid throughout the fermentation period, with only slight fluctuations. The findings of the present study are consistent with those of Somdee et al. [49], who also reported an increase in the amino acid content of rice bran during solid-state fermentation, from 1065 µg/g in the unfermented sample to 1805.87 µg/g on the fifth day of fermentation, followed by a decline to 1523.56 µg/g during the later stages of fermentation. Also, it was stated that the increase in amino acid content during the early stages of fermentation may indicate enhanced protein hydrolysis. In contrast, the subsequent decrease may be associated with further metabolic utilization of the released amino acids. Additionally, the increase in amino acid content during the fermentation may be associated with enhanced protein accessibility. Overall, the results suggest that solid-state fermentation influenced the amino acid composition of black gram, with the highest total amino acid content observed on the sixth day of fermentation.

#### 3.5.2. Fourier Transform Infrared Spectroscopy

**Figure 4:**
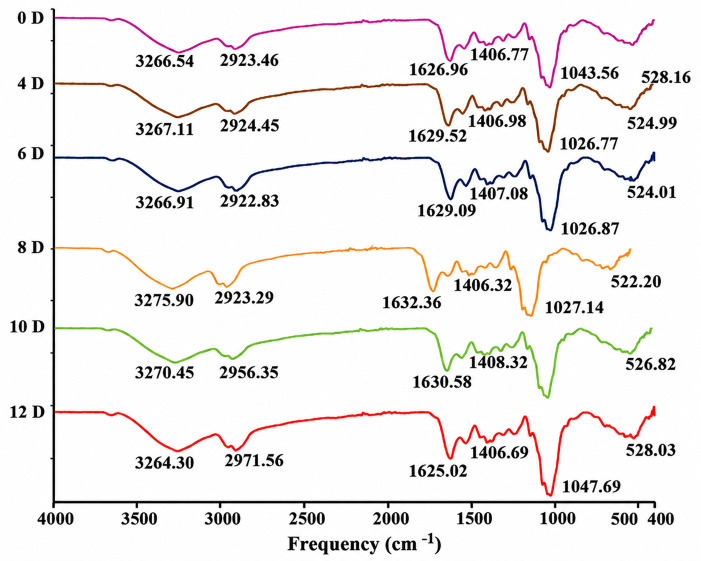
Fourier transform infrared spectroscopy of black gram fermented using *Aspergillus awamori* over 12 days (0, 4, 6, 8, 10, and 12 days).

The FTIR spectra of unfermented and fermented black gram flour are presented in Figure 4. The broad absorption band at 3264–3276 cm^−1^ is attributed to O–H and N–H stretching vibrations, while the band at 2922–2972 cm^−1^ corresponds to C–H stretching vibrations of aliphatic groups. The absorption band at 1625–1632 cm^−1^ is assigned to the amide I region, primarily associated with C=O stretching in proteins, whereas the band at 1406–1408 cm^−1^ is assigned to C–H bending and symmetric COO^−^ stretching vibrations. The band at 1026–1048 cm^−1^ is attributed to C–O and C–O–C stretching vibrations of carbohydrates, and the band at 522–528 cm^−1^ corresponds to skeletal vibrations in the fingerprint region. Overall, the FTIR spectra of fermented samples retained the characteristic absorption bands of black gram flour, although slight shifts in peak positions were observed during fermentation. These spectral variations may indicate modifications in the molecular environment of the flour components during fermentation. In contrast, the absence of major changes in the overall spectral pattern suggests that the principal functional groups remained largely unchanged.

### 3.6. Mineral Content

**Figure 5:**
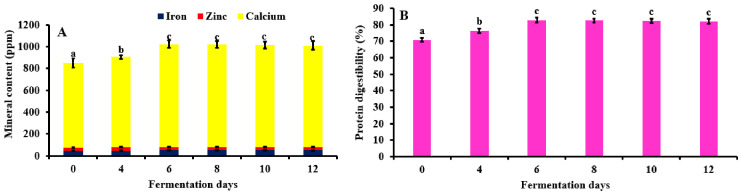
(**A**) Mineral content (**B**) In vitro digestibility of black gram fermented using *Aspergillus awamori* over 12 days (0, 4, 6, 8, 10, and 12 days). The results were expressed as the mean ± standard deviation (*n* = 3), and error bars represent the standard deviation from the mean values. In contrast, different lowercase letters above each bar indicate significantly different values (*p* < 0.05) within samples, as determined by one-way ANOVA followed by Duncan’s post hoc test for multiple comparisons.

As illustrated in Figure 5A, significant increases (*p* ≤ 0.05) in the concentrations of iron, zinc, and calcium were observed up to day 6 of fermentation. Iron concentration rose from 45.46 ppm (day 0) to 50.1 ppm, zinc from 29.5 to 31.40 ppm, and calcium from 775.16 to 940.56 ppm. However, after day 6, a non-significant decline in mineral concentration was observed (*p* ≥ 0.05). By day 12, iron concentration reduced marginally to 49.70 ppm, zinc to 30.8 ppm, and calcium to 927.56 ppm. Our results were consistent with the findings of Chawla et al. [30], who observed increased mineral content in black-eyed flour after solid-state fermentation.

### 3.7. In Vitro Digestibility

As shown in Figure 5B, protein digestibility (PD) of the sample increased notably during the early days of fermentation. The PD value increased significantly (*p* ≤ 0.05) from 70.86 ± 1.10% on day 0 to 76.41 ± 1.35% on day 4, reaching its highest level of 82.89 ± 1.42% on day 6. After day 6, a non-significant decrease (*p* ≥ 0.05) in PD was observed at 82.63 ± 1.08% on day 8, 82.35 ± 1.36% on day 10, and 82.12 ± 1.39% on day 12. The improvement in protein digestibility may be attributed to the reduction of antinutritional factors during fermentation, indirectly increasing the accessibility of proteins by enzymes, and this, in turn, increases the protein digestibility. The proteolytic activity of Aspergillus awamori during fermentation [50]. Similarly, Azeez et al. [51] observed a significant improvement in the in vitro protein digestibility of finger millet flour following solid-state fermentation, which is consistent with the findings of the present study. Based on the comparative evaluation of functional properties, structural characteristics, and in vitro protein digestibility, the six-day fermented black gram exhibited the most favorable overall characteristics among the fermentation periods investigated. Therefore, the six-day-fermented black gram was selected for incorporation into whole-wheat flour to fortify the product and for further analysis.

### 3.8. Fermented Black Gram Powder Fortified Flour

#### 3.8.1. Nutritional Properties of Flour

As shown in Table 3, incorporating black gram flour into whole wheat flour significantly improved the nutritional composition of the composite flours. Protein content increased with increasing levels of black gram flour due to its higher protein content compared with wheat, thereby enhancing the overall nutritional quality of the blends. Moisture content decreased significantly, potentially contributing to improved storage stability. Ash and crude fiber contents also increased, reflecting the higher mineral and dietary fiber contents of black gram flour. In contrast, fat content remained statistically unchanged, indicating that the level of fortification had little effect on the lipid content of the composite flours. Carbohydrate content decreased significantly because increases in protein, ash, and fiber reduced the relative proportion of carbohydrates. Overall, the results presented in Table 3 demonstrate that black gram flour fortification effectively enhances the nutritional profile of whole wheat flour, making it a promising ingredient for developing protein- and fiber-enriched bakery products.

#### 3.8.2. Water and Oil Holding Capacity of Flour

**Figure 6:**
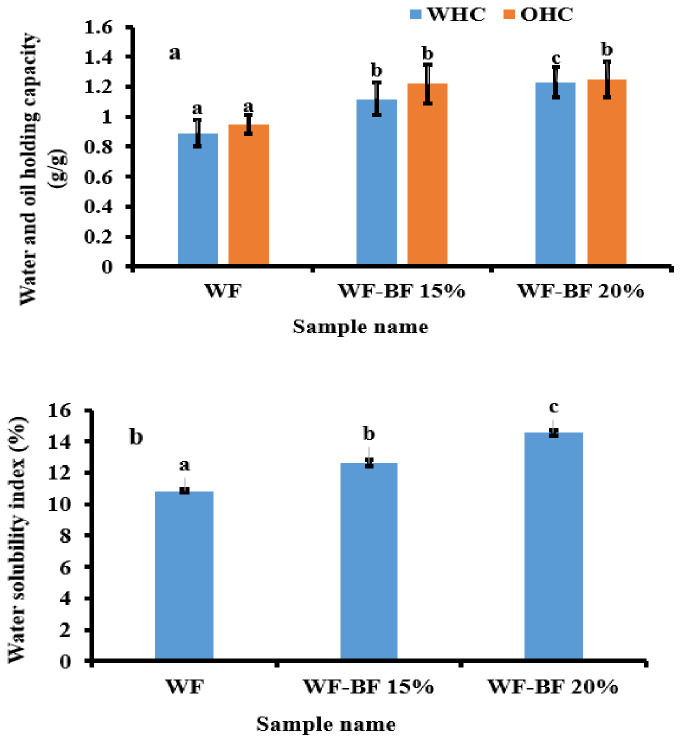
(**a**) Water and oil-holding capacity (**b**) and water solubility index of WF (whole wheat flour), WF–BF15% (wheat flour fortified with 15% black gram flour) and WF–BF20% (wheat flour fortified with 20% black gram flour). The results were expressed as the mean ± standard deviation (*n* = 3), and error bars represent the standard deviation of the mean. In contrast, different lowercase letters above each bar indicate significantly different values (*p* < 0.05) within samples, as determined by one-way ANOVA followed by Duncan’s post hoc test for multiple comparisons.

Figure 6a shows the water-holding capacity (WHC) and oil-holding capacity (OHC) of whole wheat flour (WF) and black gram flour-fortified composite flours (WF–BF15% and WF–BF20%). A significant increase in WHC was observed with increasing levels of fermented black gram flour incorporation, as indicated by different superscript letters (*p* < 0.05). This improvement may be attributed to the higher protein and dietary fiber content of fermented black gram flour. Proteins and fiber contain hydrophilic groups such as hydroxyl, carboxyl, and amino groups, which can interact with water through hydrogen bonding and enhance water retention within the flour matrix. Fermentation may also partially modify cell-wall polysaccharides and protein structures, exposing additional water-binding sites and thereby improving hydration capacity. Similarly, OHC increased significantly in the fortified flours compared with WF. The increase in OHC may be associated with the greater protein fraction in the composite flour, as protein side chains and exposed non-polar groups can interact with oil molecules. Fermentation-induced structural changes may further expose hydrophobic regions of proteins, improving their ability to entrap or bind oil. However, no significant difference in OHC was observed between WF–BF15% and WF–BF20%, as indicated by the same superscript letter (*p* > 0.05). This suggests that oil-binding sites may have reached a saturation level at 15% incorporation, and further addition of fermented black gram flour did not produce a statistically meaningful improvement. Overall, fermented black gram flour fortification improved both water- and oil-binding properties of the composite flour. However, increasing the fortification level from 15% to 20% produced an additional significant improvement only in WHC, while OHC remained statistically comparable between the two fortified formulations. From a product-development perspective, higher WHC may improve dough hydration, softness, and yield, whereas improved OHC may contribute to better flavor retention and mouthfeel in cereal–legume-based food products.

#### 3.8.3. Water Solubility Index of Flour

Figure 6b shows the water solubility index (WSI) of whole wheat flour (WF) and black gram flour-fortified composite flours (WF–BF15% and WF–BF20%). The WSI increased significantly with increasing levels of black gram flour incorporation, as indicated by different superscript letters (*p* < 0.05). WF–BF15% exhibited a significantly higher WSI than WF, while WF–BF20% showed the highest WSI among all the samples. Overall, black gram flour fortification significantly increased the water solubility index of the composite flours.

#### 3.8.4. In Vitro Digestibility of Flour

**Figure 7:**
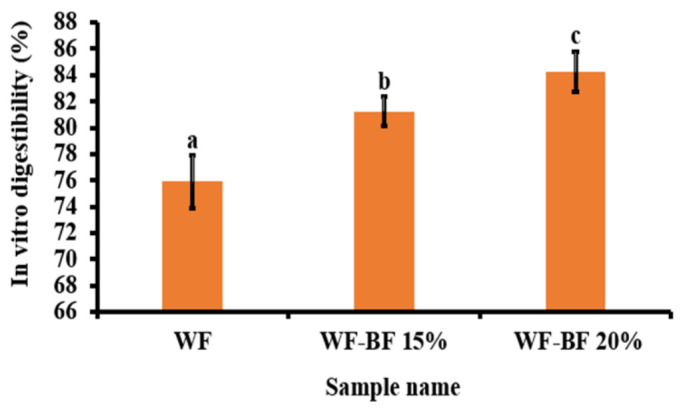
In vitro digestibility of WF (Wheat flour), WF–BF15% (wheat flour fortified with 15% black gram flour), and WF–BF20% (wheat flour fortified with 20% black gram flour). The results were expressed as the mean ± standard deviation (*n* = 3), and error bars represent the standard deviation of the mean. In contrast, different lowercase letters above each bar indicate significantly different values (*p* < 0.05) within samples, as determined by one-way ANOVA followed by Duncan’s post hoc test for multiple comparisons.

As shown in Figure 7, WF (wheat flour) exhibited the lowest in vitro protein digestibility, whereas incorporating fermented black gram flour resulted in a significant increase (*p* < 0.05) in digestibility. WF–BF15% showed significantly higher digestibility than WF, whereas WF–BF20% showed a further significant improvement over WF–BF15%. Among all samples, WF–BF20% exhibited the highest in vitro protein digestibility, indicating a concentration-dependent enhancement as the level of fermented black gram flour increased. The improved in vitro protein digestibility of the composite flours may be associated with the incorporation of fermented black gram flour, which previously exhibited higher protein digestibility, increased amino acid content, and reduced levels of antinutritional factors such as phytic acid and tannins. These changes may have collectively contributed to the improved digestibility of the composite flour.

## 4. Conclusions

The integration of controlled fermentation enhances the nutritional quality, functional characteristics, and biochemical composition of legumes. During fermentation, microbial enzyme activity modified the macromolecular components, resulting in measurable changes in the compositional and functional attributes of black gram. Among the fermentation periods evaluated, the six-day fermented sample exhibited the highest values for several nutritional and functional parameters under the experimental conditions of the present study. Beyond this point, some parameters plateaued or declined marginally, suggesting that extended fermentation may not confer additional functional advantages. The functional changes achieved through fermentation were reflected in the performance of fermented black gram when incorporated into wheat flour formulations. The WF–BF composite flours exhibited measurable changes in water-holding capacity, oil-holding capacity, and water solubility index, demonstrating the applicability of fermented black gram as a functional ingredient in composite flour systems. These results support the use of fermentation-treated black gram for product formulation. The present study focused on the nutritional and functional characteristics of the composite flours; therefore, sensory attributes, product quality, and processing performance were not evaluated and warrant further investigation.

## 5. Limitations

The present study demonstrated the potential of solid-state fermentation using *Aspergillus awamori* to improve the nutritional, functional, and antioxidant properties of black gram. However, certain limitations should be acknowledged. The study was conducted using a single microbial strain, and the effects of other microorganisms or mixed-culture fermentations were not investigated. Although significant changes in nutritional and functional properties were observed, the specific enzymatic activities responsible for these modifications were not directly measured. Similarly, the individual bioactive compounds contributing to the enhanced antioxidant activity were not identified. The experiments were performed under controlled laboratory conditions, which may differ from large-scale industrial fermentation systems. Furthermore, protein digestibility was assessed using an in vitro method, which may not fully reflect the physiological conditions of the human gastrointestinal tract. In addition, sensory attributes, storage stability, and consumer acceptance of the fortified flour products were not evaluated and require further investigation.

## Figures and Tables

**Table 1:** Proximate analysis of non-fermented black gram.

Proximate Analysis	Content (%)
Protein content	24.13 ± 0.25
Moisture	10.49 ± 0.08
Ash	3.28 ± 0.17
Fat	1.44 ± 0.05
Crude fiber	5.13 ± 0.16
Carbohydrate	55.53 ± 0.57

The results are expressed as mean ± standard deviation (*n* = 3). Error bars represent the standard deviation of the mean values.

**Table 2:** Changes in amino acid content (g/100 g protein) of black gram during fermentation with *Aspergillus awamori* over 12 days (0, 4, 6, 8, 10, and 12 days).

Amino Acid	0 Day	4 Day	6 Day	8 Day	10 Day	12 Day
Essential Amino Acids						
Histidine	3.35	3.45	3.73	3.59	3.45	3.20
Isoleucine	3.58	3.75	3.98	3.84	3.72	3.36
Leucine	6.5	7.13	7.01	6.85	6.30	6.28
Lysine	6.13	6.33	6.4	6.11	6.12	5.91
Methionine	0.99	1.12	1.17	1.11	0.99	0.77
Phenylalanine	8.51	9.06	9.15	9.19	8.45	8.07
Threonine	3.21	3.39	3.76	3.47	3.36	2.99
Valine	3.90	4.37	4.30	4.27	4.16	3.68
**Non-Essential Amino Acids**						
Alanine	3.96	4.39	4.53	4.29	4.18	3.75
Arginine	5.35	5.96	6.13	5.56	5.26	5.13
Aspartic acid	8.40	9.43	9.60	8.40	8.24	8.18
Cysteine	0.88	1.16	1.26	1.20	1.09	0.66
Glutamic acid	19.76	20.20	20.19	19.10	18.21	19.54
Glycine	3.19	3.56	3.82	3.61	3.50	2.98
Proline	4.21	4.70	4.98	4.79	4.68	3.99
Serine	4.47	4.81	5.12	4.89	4.78	4.25
Tyrosine	2.32	2.93	3.11	2.84	2.73	2.10
**Total amino acid content (g/100 g)**	**88.71**	**95.74**	**98.24**	**93.11**	**89.22**	**84.84**

**Table 3:** Nutritional profile of composite flour.

	WF	WF-BF15%	WF-BF20%
Protein content	12.86 ± 0.16 ^a^	14.58 ± 0.16 ^b^	15.22 ± 0.34 ^c^
Moisture	11.22 ± 0.33 ^c^	9.22 ± 0.33 ^b^	8.85 ± 0.17 ^a^
Ash	1.55 ± 0.10 ^a^	1.76 ± 0.17 ^b^	1.82 ± 0.13 ^b^
Fat	2.33 ± 0.23 ^a^	2.37 ± 0.29 ^a^	2.39 ± 0.13 ^a^
Crude Fiber	1.12 ± 0.11 ^a^	1.45 ± 0.28 ^b^	1.96 ± 0.19 ^c^
Carbohydrate	70.48 ± 0.77 ^b^	69.82 ± 0.73 ^b^	68.76 ± 0.31 ^a^

Data are presented as mean ± standard deviation (*n* = 3). Statistical significance between WF (Whole wheat flour), WF–BF15% (wheat flour fortified with 15% black gram flour), and WF–BF20% (wheat flour fortified with 20% black gram flour) was assessed using one-way ANOVA, followed by Duncan’s post hoc test for multiple comparisons. Different superscript letters within the same row indicate significant differences (*p* < 0.05).

## Data Availability

The data presented in this study are available on request from the corresponding author. The data are not publicly available due to privacy restrictions.
